# Supplementary material for: DMRforPairs: identifying Differentially Methylated Regions between unique samples using array based methylation profiles
Source: BMC Bioinformatics. 2014 May 15;15:141. doi: 10.1186/1471-2105-15-141 (PMC4046028; doi:10.1186/1471-2105-15-141)
Supplement: Additional file 2 — DMRforPairs output for the comparison of A431-MCF7 and NA17018-NA17105. Please start from the HTML files in each folder. Available via the BMC Bioinformatics website. [file 1471-2105-15-141-S2.zip › 1394847754114233_MOESM2_ESM/A431_MCF7/figures/5890.pdf]

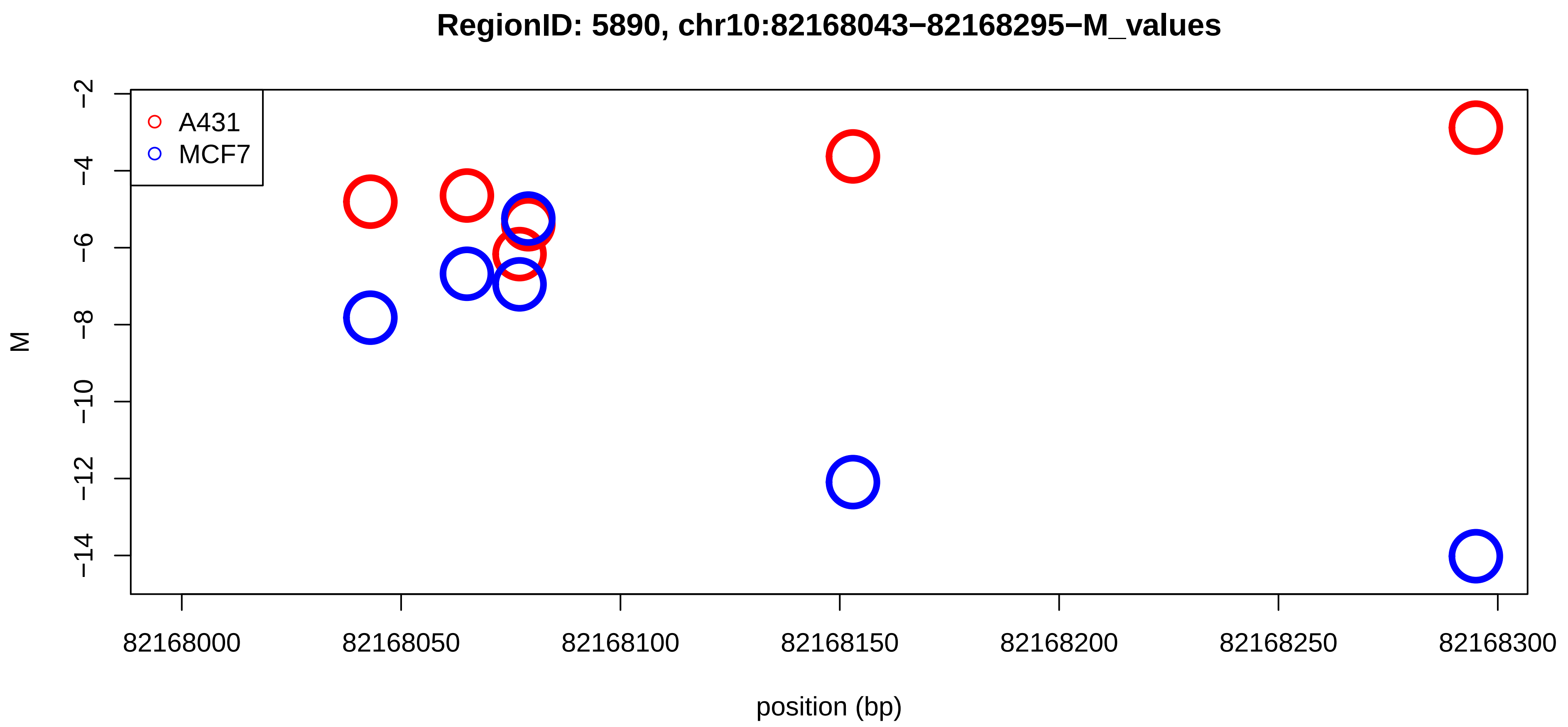
RegionID: 5890, chr10:82168043−82168295−M_values
A431
MCF7
M
−2
−4
−6
−8
−10
−12
−14
82168000
82168050
82168100
82168150
82168200
82168250
82168300
position (bp)

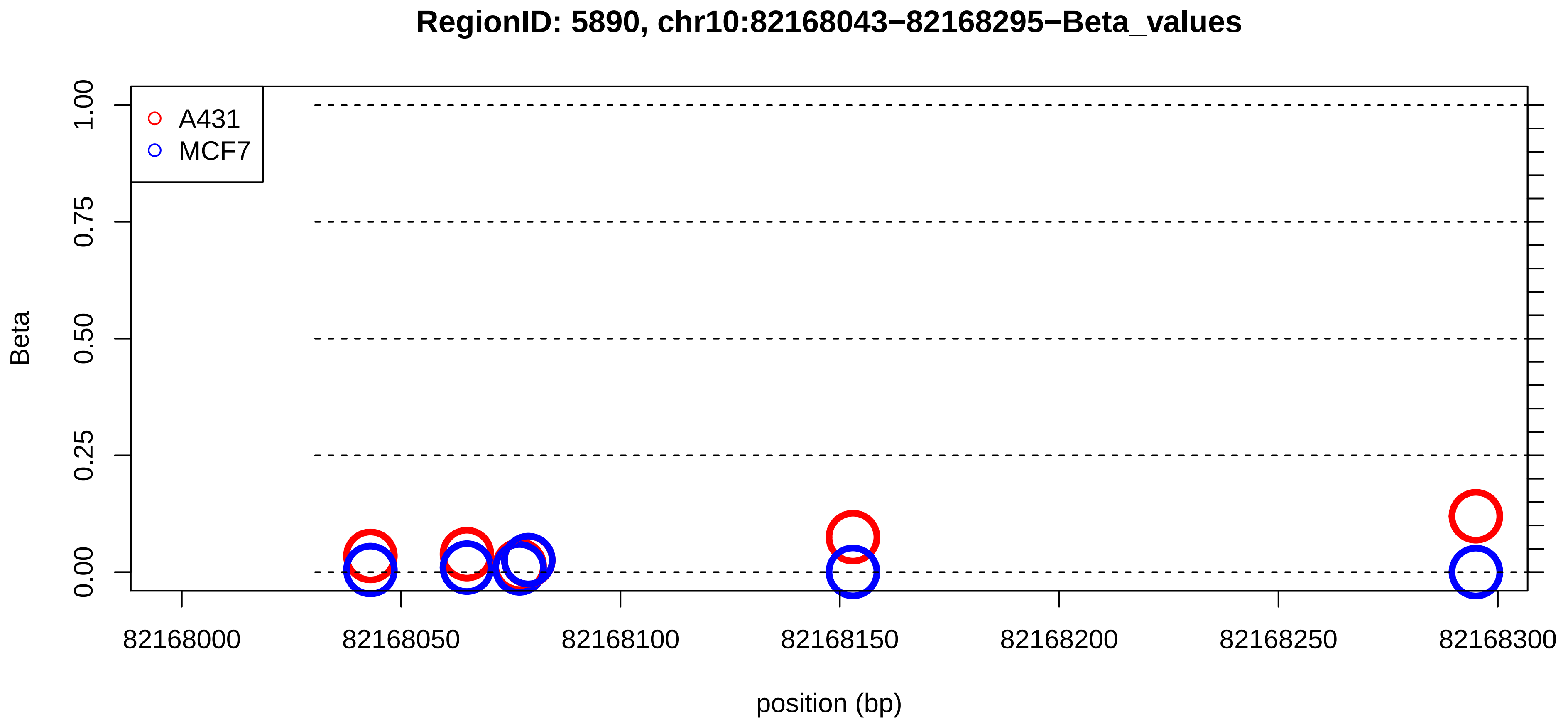
RegionID: 5890, chr10:82168043−82168295−Beta_values
A431
MCF7
Beta
1.00
0.75
0.50
0.25
0.00
82168000
82168050
82168100
82168150
82168200
82168250
82168300
position (bp)

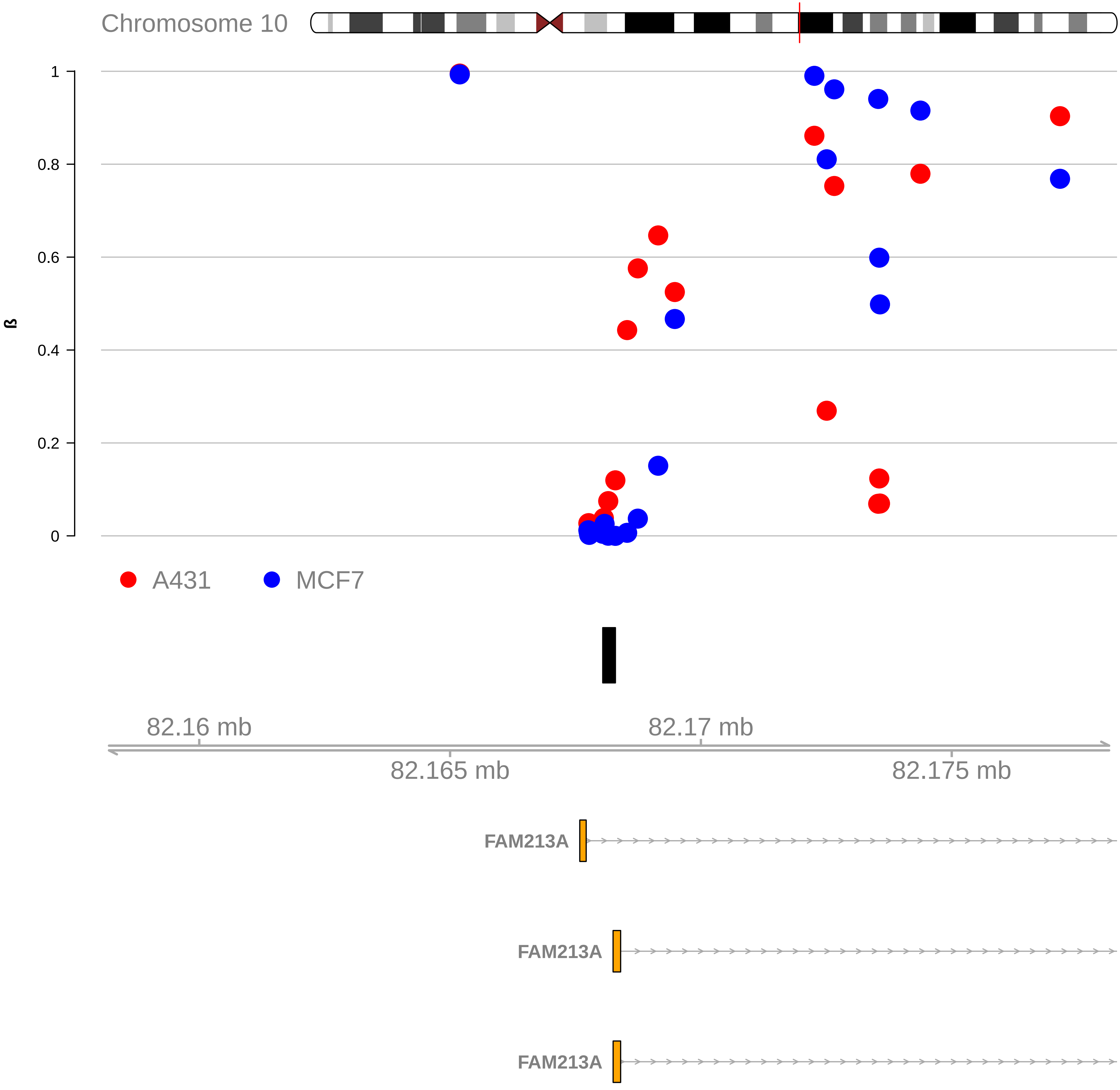

Chromosome 10
1
0.8
0.6
0.4
0.2
0
ß
A431
MCF7
82.16 mb
82.17 mb
82.165 mb
82.175 mb
FAM213A
FAM213A
FAM213A
